# Utility of self-reported mental health measures for preventing unintentional injury: results from a cross-sectional study among French schoolchildren

**DOI:** 10.1186/1471-2431-14-2

**Published:** 2014-01-08

**Authors:** Aymery Constant, Judith Dulioust, Ashley Wazana, Taraneh Shojaei, Isabelle Pitrou, Viviane Kovess-Masfety

**Affiliations:** 1 EHESP School of Public Health, Avenue du Prof Leon Bernard, Rennes, France; 2 Direction de l’Action Sociale, de l’Enfance et de la Santé, Quai de la Rapée, Paris, France; 3 Department of Psychiatry, Jewish General Hospital, Chemin de la Côte-Sainte-Catherine, McGill University, Montreal, Quebec, Canada; 4 Centre hospitalier Paul Guiraud, Rue Dispan, Villejuif, France; 5 EA 4069 Paris Descartes University, Rue de l’école de médecine, Paris, France

**Keywords:** ADHD, Injury, School children, Screening, Infant mental health, Self-report

## Abstract

**Background:**

Identify children at-risk of having mental health problems is of value to prevent injury. But the limited agreement between informants might jeopardize prevention initiatives. The aims of the present study were 1) to test the concordance between parents and children reports, and 2) to investigate their relationships with parental reports of children’ unintentional injuries.

**Methods:**

In a population-based sample of 1258 children aged 6 to 11, the associations between child psychopathology (using the Dominic Interactive and the Strengths and Difficulties Questionnaire) and unintentional injuries in the past 12 months were examined in univariate and multivariate models.

**Results:**

As compared to children, parents tended to overestimate behavior problems and hyperactivity/inattention, and underestimate emotional symptoms. Unintentional injury in the last 12-month period was reported in 184 out of 1258 children (14.6%) and multivariate analyses showed that the risk of injury was twice as high in children self-reporting hyperactivity/inattention as compared to others. However this association was not retrieved with the parent-reported instrument.

**Conclusion:**

Our findings support evidence that child-reported measures of psychopathology might provide relevant information for screening and injury prevention purposes, even at a young age. It could be used routinely in combination with others validated tools.

## Background

For the assessment of childhood psychopathology, there is no measurement for which the accuracy (validity) and precision (reliability) are sufficiently high to give indisputable evidence, either for clinical care, research, or screening purposes [1]. Accordingly, assessment using data from multiple informants (e.g., children themselves; their parents, teachers, and clinicians) is highly recommended to improve decision making on diagnostic and intervention issues [2]. However, convergence of the data is rarely achieved. Recent evidence indicated that data from teachers and parents might disagree in their reports because of differing expertise [3]. Additionally, there is scepticism about children’s reliability [4]. Furthermore, when screening children who did not yet have behavioral symptoms, both parent and teacher measures resulted in substantial misclassification errors [5].

This issue might be of importance for prevention initiatives towards schoolchildren. Indeed, mental health problems such as Attention Deficit Hyperactivity Disorder (ADHD), Conduct Disorder (CD) and Oppositional Defiant Disorder (ODD) might increase the risk of injury among children [6-12]. Byrne et al. [13] found that preschool-aged children with ADHD exhibit behaviours (e.g., inattention and impulsivity) which place them at a higher risk of serious injury requiring a visit to the emergency department. This is explained by a reduced attentional monitoring required to complete daily activities without danger [14] and a greater difficulty in recognizing hazards and evaluating risks [15]. Others Significant risk factors include demographic, family, and environmental variables [16]. Unintentional injuries are more common in boys as compared to girls, and are associated with lower Socio Economic Status [17], neighbourhood deprivation [18], and rural area of residence [19].

Unintentional injuries are the leading cause of childhood morbidity and mortality in elementary school children [20-22]. To prevent such severe health issues, it is valuable to identify children and adolescents at-risk of having mental health problems and those who would most benefit from more in-depth assessment. However, there is little or no data on this topic, and misclassification errors might jeopardize prevention initiatives. The aims of the present study were 1) to test the concordance between parents and children reports, and 2) to investigate their relationships with parental reports of children‘s’ unintentional injuries in the last 12-month period.

## Methods

### Study sample

To ensure representativeness across the 1856 schools of the area (approximately 296,257 pupils), a stratified 2-level probability sample was selected with randomization of 100 primary schools and 25 children per school (five from each of grades 1 to 5). Randomization was stratified on the following school characteristics: public/private, rural/urban, and Deprived School Areas (DSA)/no DSA. Of the 100 primary schools selected, 99 agreed to participate. Contacts were attempted for 2,341 children. Further details on the sampling procedure and methods can be found in previous reports [23].

### Ethical approval and data collection

The research plan was approved by the French national Committee on Ethics (CNIL). Informational letters about the objectives of the study, refusal forms, and a postage-paid return envelope were sent to parents of the selected children. Anonymity was guaranteed, and participants were able to withdraw from the study at any time.

### Self-reported child measure

The Dominic Interactive (DI) is an interactive self-report instrument for young children (6 years and older), consisting of 91cartoons depicting a child named Dominic/Dominique with a feeling, a thought or an act. A voice-over describes the symptom and asks the child if she or he acts, feels or thinks similarly. The DI generates a probability diagnosis towards the following seven mental health disorders: specific phobias (SPh), major depression, (MDD), separation anxiety (SAD), generalized anxiety disorders (GAD), hyperactivity/inattention, Oppositional Defiant Disorder (ODD), and Conduct problem (CP). The DI has been validated by several studies [24-28]. Loney et al. found that the reliability of the DI is better than those of structured interviews for young children [29]. The psychometric properties of the French version of the DI are satisfactory [30]. Children completed the DI on a computer station at school under the supervision of a research assistant.

### Reported parental measures

The Strengths and Difficulties Questionnaire (SDQ) provides diverse measures of child mental health problems (emotional symptoms, hyperactivity/inattention, conduct problems, peer relationship problems and prosocial behavior (5 items)) [30,31]. The SDQ is shorter than alternative measures of child psychopathology and has been used to study injured children [10]. It has been extensively evaluated and is reliable and valid [32]. Good psychometric properties of the French version of the SDQ have been reported in an epidemiological sample of 1,400 youths [33] and in this sample [23].

### Parental reports of injury

Sociodemographic data, parents were asked “in the past 12 months, did your child incur an accident requiring either a contact with a physician or a visit to the hospital?”. If yes, they were asked to provide details about the most recent injury, including where (e.g., home, school) and how (e.g., falling, poisoning, etc.) the injury occurred. Information on the anatomical site of the injury (e.g., head, limbs), and the type of injury (e.g., burn, fracture) were also collected. Injuries were coded according to the International Classification of Diseases, Ninth edition (N codes 800–994).

### Data analysis

Parents’ reports of child’s injury in the last 12-month and others categorical variables were expressed as a percentage (%) and compared with Chi square tests. A mean score was calculated for each subscale of the DI and the SDQ, and validated cut-off limits were applied to classify children as regards to the presence of a mental health problem (yes/ borderline/no). In order to obtain conservative estimates, borderline scores were considered as an absence of psychopathology. Kappa coefficients were computed to estimate the level of agreement between DI and SDQ. Since our study outcome was binomial (injuries: yes/no), we used logistic regression models to estimate the odds ratios of reported unintentional injury as a function of emotional and behavioral problems, separately for each tool. In order to address the potential confounding effect of each factor, we used two series of models. First, the association of each mental health problem with the risk of reporting injury was assessed separately (model 1; one model per factor, adjusted on male gender, parental unemployment; living in rural area and school located in a deprived area ). All variables associated (p value <0.10) with the risk of reporting injury in model 1 were included in a single multivariate analysis (model 2), with adjustment on male gender, parental unemployment; living in rural area and school located in a deprived area. The analyses were carried out with SPSS version 19.

## Results

a) Socio-demographic characteristics of the study sample

Of the 2,341 eligible parents, 462 (19.7%) refused to participate and 531 (22.7%) did not return the questionnaire. Complete parent and child data were available for 1258 children (males: 50.2%), with a mean age of 8.2 years (Standard deviation SD = 1.50). Most children were born in France (95.2%), with 92.3% of them living in urban areas and 12.6% with an unemployed parent (Table 1). To assess a possible response bias, we compared responding and nonresponding parents by school area and parental socio-economic status and did not find any statistical differences.

b) Presence of unintentional injuries

During the last 12-month period, 184 (14.6%) children sustained unintentional injuries (Table 2). Boys were more frequently injured as compared to girls (17.4% vs. 11.9%, respectively; p < 0.004). Most injuries occurred at school (46.7%). They occurred mostly during sports activities (51.9%) and following accidental falls (27.0%). Injuries were mostly sprains (29.3%), wounds/cuts (28.1%) and fractures/dislocations (23.0%), located on the limbs (59.2%). A minority of unintentional injuries (8.6%) led to hospitalization.

a) Reliability between parents and children reports

1) Emotional symptoms

Emotional symptoms were reported in 10.8% of children by parents using the SDQ, while 17.4% of children self-reported at least one emotional symptom (MDD, GAD, SpH, SAD) using the DI (Table 3). The value for Kappa is 0.04, indicating a very low level of concordance between parent- and child-reported measures. A Cross-Tables analysis indicates that 189 children (15%) reporting emotional symptoms with the DI were considered normal by parents using the SDQ (Table 4).

2) Hyperactivity/inattentionHyperactivity/inattention was reported in 12.2% of children by parents and self-reported by 4.5% of children using the DI. The value for Kappa is 0.04, indicating a very low level of agreement. Cross-Tables statistics indicates that 138 children (11.0%) considered as having hyperactivity/inattention with the SQD were considered normal with the DI.

3) Behavioral problems

Conduct problems were reported in 11.8% of children by parents using the SDQ, while 8.3% of children self-reported at least one conduct problem (CD, ODD) using the DI, the value for Kappa is 0.10, indicating a poor level of agreement. A Cross-Tables analysis indicates that 125 (9.9%) children considered as having conduct problem with the SQD were considered normal with the DI.

The associations between injury risk and scores on the DI and the SDQ sub-scales are reported in Table 5. In univariate analysis, the likelihood of injury was higher in children with self-reported hyperactivity/inattention, GAD, ODD and Pro-social difficulties as compared to others. In multivariate analysis, the likelihood of injury was higher in children with self-reported hyperactivity/inattention only. No significant association was found between the parent-reported SDQ sub-scales and unintentional injuries.

**Table 1:** Sociodemographic characteristics of the study sample (N = 1258)

**Variables**		**N (%)**
Age	6-8 years	753 (59.8)
	9-11 years	505 (40.2)
Gender	Girls	627 (49.8 )
	Boys	631 (50.2)
Parental education	< High school	462 (36.7)
	≥ High School	796 (62.3)
Parental unemployment	No	1101 (87.4)
	Yes	157 (12.6)
Demographic area	Urban	1160 (92.3)
	Rural	98 (7.7)
Deprived school area	No	1133 (90.1)
	Yes	125 (9.9)

**Table 2:** Characteristics of the 184 unintentional injuries of children aged 6 to 11 from a French representative sample (N = 1258)

	**N (%)**
**Place of occurrence**^**1**^	
School	84 (46.7)
Home	33 (18.3)
Sport field	22 (12.2)
Street	16 (8.9)
Other	29 (13.9)
**Activity during injury**^**1**^	
Sports	94 (51.9)
Falls	47 (27.0 )
Non motor-vehicle pedal cycle	17 (9.4 )
Hit by object	17 (9.3 )
Cutting or piercing	11 (6.1 )
**Injured part**^**1**^	
Limb	114 (59.2 )
Face/	47 (25.0 )
Head	21 (11.2 )
Others (Chest, abdomen, back)	25 (13.9 )
**Lesion type**^**1**^	
Sprain	54 (29.3 )
Wound, cut	48 (28.1 )
Fracture, dislocation	43 (23.0 )
Contusion	36 (19.0 )
Head injury	19 (10.3 )
Burn	5 (2.3 )
Poisoning, bite	4 (2.3 )
**Hospitalization**	8.6 (16)

^**1**^several responses were allowed.

**Table 3:** Prevalence of mental health problems, by gender, according to parent and child report, in a representative sample of children aged 6–11 years old (N = 1258)

	**All**	**Boys (%)**	**Girls (%)**	**P value**
**Measures**				
**Emotional symptoms**				
Parent report – SDQ	10.8	10.2	11.0	**0.28**
Child report-DI				
GAD	5.6	4.5	6.6	0.06
SAD	8.4	7.7	9.2	**0.19**
MDD	4.1	4.5	3.6	**0.27**
SPh	7.4	6.1	8.7	<0.05
At least one	17.4	15.3	19.5	<0.04
**Hyperactivity/inattention**				
Parent report – SDQ	12.2	16.1	8.4	<0.001
Child report - DI	4.5	6.1	2.8	<0.01
**Behavior problems**				
Parent report – SDQ				
Conduct problems	11.8	14.5	9.2	0.002
Peer problems	14.8	15.8	13.8	**0.17**
Pro-social difficulties	2.1	3.0	1.1	0.001
Child report DI				
CD	4.6	7.5	1.7	<0.001
ODD	5.0	5.6	4.4	**0.21**
At least one	8.3	10.7	5.9	0.001

Dominic Interactive (DI) symptom sub-scales: GAD - Generalized Anxiety Disorder, SAD - Separation Anxiety Disorder, MDD - Major Depressive Disorder, Sph- Specific Phobia ADHD- Attention Deficit-Hyperactivity Disorder, ODD - Oppositional Defiant Disorder, CP - Conduct Problem, SDQ- Strengths and Difficulties Questionnaire.

**Table 4:** Concordance in mental health screening between parent and children’ reports

	**Type of mental health problems assessed both by DI and SDQ**		
	**Emotional symptoms**	**Hyperactivity-inattention**	**Behavior problems**
**Presence of mental health problems**	N (%)	N (%)	N (%)
None, according to SDQ and DI	933 (74.2%)	1064 (84.6%)	1029 (81.8%)
Yes, according to SDQ only	106 (8.4%)	138 (11.0%)	125 (9.9%)
(Parent-reported measure)			
Yes, according to DI only	189 (15.0%)	40 (3.2%)	80 (6.4%)
(Child self-reported measure)			
Yes, according to SDQ and DI	30 (2.4%)	16 (1.2%)	24 (1.9%)
Kappa value	0.04	0.04	0.10

Note: SDQ: Strengths and Difficulty Questionnaire; DI: Dominic Interactive.

**Table 5:** Association between unintentional injuries and parents’ and children’ reports of mental health problems, determined by logistic regression

**Variables**	**Univariate model; adjusted estimates**						**Multivariate model; adjusted estimates**					
	**B**	**SE**	**Wald**	**df**	**p**	**Exp (B)**	**B**	**SE**	**Wald**	**df**	**p**	**Exp (B)**
**Parent report – SDQ**												
Emotional symptoms	0.12	0.25	0.24	1	0.62	1.13						
Hyperactivity-inattention	0.32	0.22	2.05	1	0.15	1.38						
Conduct problems	0.19	0.23	0.63	1	0.42	1.21						
Peer problems	0.24	0.22	1.24	1	0.26	1.27						
Pro-social difficulties	0.79	0.46	2.97	1	0.08	2.19	0.70	0.46	2.27	1	0.13	2.02
**Child report - dominic interactive**												
GAD	0.73	0.29	6.15	1	0.02	2.08	0.45	0.33	1.89	1	0.17	1.57
SAD	0.34	0.27	1.61	1	0.20	1.41						
MDD	0.53	0.35	2.25	1	0.13	1.70						
SPh	0.13	0.31	6.17	1	0.67	1.14						
**Hyperactivity/inattention**	**1.10**	0.30	13.7	1	0.001	3.01	0.88	0.34	6.53	1	0.01	2.41
CD	0.56	0.32	2.97	1	0.08	1.75	0.19	0.36	0.27	1	0.60	1.21
ODD	0.59	0.31	3.60	1	0.06	1.80	0.04	0.37	0.01	1	0.92	1.04
**Male gender**							0.41	1.67	5.88	1	0.01	1.5
**Parental unemployment**							0.32	0.23	1.91	1	0.17	1.38
**Deprived neighborhood**							−0.42	0.31	1.86	1	0.17	0.65
**Rural area**							0.38	0.21	3.27	1	0.07	1.46

Note: Dominic Interactive symptom sub-scales: GAD - Generalized Anxiety Disorder, SAD - Separation Anxiety Disorder, MDD - Major Depressive Disorder, Sph- Specific Phobia ADHD- Attention Deficit-Hyperactivity Disorder, ODD - Oppositional Defiant Disorder, CD - Conduct Disorder.

SDQ- Strengths and Difficulty Questionnaire.

SE = standard error; df = degree of freedom; Exp(B) = exponentiation of the B coefficient (Odds ratios).

## Discussion

Findings from the present study showed that parent- and child-reported measures of psychopathology were not concordant. Estimates of behavior problems/hyperactivity/inattention were higher in parent’s reports compared to children’s reports, while those of emotional symptoms were higher in children compared to parents. Multivariate analyses showed that the risk of injury was twice as high in children reporting hyperactivity/inattention as compared to others, a result in line with previous studies [6-8]. However this association was not retrieved with the parent-reported instrument. Our findings support the evidence that child-reported measures of psychopathology might provide relevant information for screening and injury prevention purposes, even at a young age. It could be used routinely in combination with others validated tools.

Both parent and children measures indicated a higher prevalence of behavior problems and a lower prevalence of emotional symptoms among boys as compared to girls. However, the concordance between children and parental estimates was poor. As compared to the children’s reports, parents seem to have minimized intrinsic problems such as anxiety, phobia or depression, and amplified extrinsic problems with visible manifestations, such as behavior problems and hyperactivity/inattention. Interestingly, such a tendency has been previously observed. In a study including schoolchildren in Canada [34], internalizing disorders were underestimated by external observers (parents and teachers) while ADHD was reported more frequently by teachers (9.8%) as compared to parents (6.9%) and children (3.8%). When it comes to anxiety, of which symptoms are quite covert, reliance on parent reporting produces lower rates of anxiety than using children alone, or in combination with other informants [35]. In a study focusing on discrepant reports where only one of the informant accounted for the presence of a child diagnosis, authors suggested that children could be better informants than parents for their internalizing disorders, because they directly experience and are quite often aware of their internal states and feelings, whereas parent might be better reporters of externalizing disorders [36].

This statement however has to be mitigated. To some degree, impulsive behaviors, intense activity, and distraction are common among children 6–11 years old. These might be interpreted as pathologic symptoms by parents, in a context where ADHD was largely mediatized. Such bias has been recently documented among specialists; this has led to ADHD over-diagnosis in the past decades, as well as significant increases in medication costs [37-39]. In addition, the prevalence of ADHD is 5.2% worldwide and 4.6% in Europe [40]. In the present study, the prevalence of hyperactivity/inattention was 4.5% according to children self-report, and 12.2% according to parental measures. Only child-reported hyperactivity/inattention was related to unintentional injury. In the absence of any clinical psychiatric assessment, there remains the possibility of misclassification errors. But these results nonetheless suggest that a tool designed to thoroughly assess children perception of their own difficulties could be of interest for screening purposes in combination with other validated tools.

When it comes to other mental health problems assessed in the study, comparing findings from the present study with other estimates is difficult, since epidemiological studies have varied substantially in the prevalence rates reported. A review including 11 studies that investigated the prevalence of DSM-III or DSM-IV anxiety, specifically in children aged under 12, indicated that the rates of diagnosis varied between 2.6% and 41.2% [35]. It must be stressed, however, that children’s reports from our study are in line with aggregated results indicating that separation anxiety is the most common individual disorder and that anxiety disorders are more common than depressive disorders [35].

This report has various strengths. The sample is a large-scale randomized French sample using strategies to ensure faithful estimates of population values; the association between unintentional injuries and child psychopathology symptoms was examined using both parent and child report; and the non-response rate was satisfactory and consistent with many cross-sectional surveys using mailed self-report questionnaires [41,42]. Although parents were asked to describe only one injury, the estimate of one-year incidence in our study (13.6%) fell within the known French range (11.4% to 15.3%) [43,44]. And the hospitalization rate in our sample was also close to that of other studies (7%-9%) [44,45]. However, parents’ alcohol consumption, poor parental supervision, deliberate injuries and injuries as a result of violence were not assessed and it was not possible to determine the causal relationship between psychopathology and unintentional injuries given the cross-sectional design of our study.

## Conclusions

Health practitioners might be reluctant for practical and ethical reasons to interview the children themselves and rely on information from adults only. Our findings however support the evidence that child-reported measures of psychopathology symptoms might provide relevant information for screening and injury prevention purposes, even at a young age. They could therefore be used routinely in combination with others validated tools.

## Competing of interest

The authors report no conflict of interest.

## Authors’ contributions

VK and IP contributed to the conception and design of the study. SJ, JD, and AW, performed the data collection. AC, AW, and VK interpreted the data and wrote the manuscript. All the authors read and approved the final manuscript.

## Pre-publication history

The pre-publication history for this paper can be accessed here:

http://www.biomedcentral.com/1471-2431/14/2/prepub
